# Effects of Combination Movement Patterns Quality and Physical Performance on Injuries in Young Athletes

**DOI:** 10.3390/ijerph18115536

**Published:** 2021-05-21

**Authors:** Dawid Koźlenia, Jarosław Domaradzki

**Affiliations:** Department of Biostructure, Faculty of Physical Education, University School of Physical Education in Wroclaw, al. I.J. Paderewskiego 35, 51-612 Wroclaw, Poland; jaroslaw.domaradzki@awf.wroc.pl

**Keywords:** injury, young athletes, movement patterns, physical performance, flexibility, strength, power, balance, prevention

## Abstract

Identifying the factors associated with the injuries is crucial to prevention, enabling apply effective methods to reduce injuries frequency. This is especially important for young athletes for whom an injury may impair development or prematurely end a sports career. Therefore, the objective of this study is to examine if the movement patterns quality and physical performance simultaneously affected injury occurrence in young athletes. The participants were 176 athletes aged 22.44 ± 1.64. The injury data were collected from the 12 months retrospective period. The functional movement screen test was conducted to assess the quality of movement patterns, and the physical performance tests were done for assessing strength, power, flexibility, and balance. Results showed relationships between movement patterns quality and flexibility with injuries. The receiver operating characteristic curve demonstrated growing injury frequency for 14 ≥ FMS and 21 cm ≥ Sit-and-reach test. Rank Transform ANOVA revealed a simultaneous effect of movement quality (*F* = 11.5361; *p* = 0.0008) and flexibility (*F* = 8.0514; *p* = 0.0050) on an injury. Post-hoc tests indicated that the group with low-quality movement patterns combined with a low level of flexibility is the most frequently injured (*p* < 0.05). It is recommended to include in training, routine exercises improving movement patterns and flexibility to prevent injuries.

## 1. Introduction

Identifying the factors associated with injury occurrence is crucial to prevention, allowing the use of appropriate and effective methods to reduce injuries. This issue is especially important for young athletes for whom an injury may impair development or even prematurely end their sports careers [1,2]. They are a particularly vulnerable group due to the increased exposure to injuries [3]. Knowledge of the conditions of injuries is a basis for effective prevention [4].

Some universal factors are an underlying injury occurrence among athletes, despite sport or sex [5,6]. However, injury factors are mainly determined singly without a complex view, which could be more accurate and reliable, implying more effective prevention methods in training. Additionally, links with an injury of them remain unclear [6]. The etiology of injuries is described through the prism of extrinsic factors, which [7] includes wrong or lack of warm-up before physical activity, lack of proper regeneration [8], or intrinsic factors [7] as an abnormal morphological structure [9], low-quality movement patterns [10,11], or the level of physical performance [12,13,14]. Some intrinsic injury factors are modifiable, as movement quality, flexibility, muscle strength, or balance. Therefore, it is needed to identify which of them are crucial in injury etiology and shaping them is effective in injury prevention.

The tool for assessing the quality of movement patterns is the functional movement screen—the FMS test [15]. The FMS test is a reliable tool characterized by high reliability [16]. A meta-analysis done by Bonazza et al. [17] indicates the ICC for intrarater reliability was 0.81 (95% CI, 0.69–0.92) and for interrater reliability was 0.81 (95% CI, 0.70–0.92). Numerous studies among athletes have indicated relationships between poor FMS scores, which indicate dysfunctional movements with a high number of injuries in retrospective terms, despite the sport type [10,11,18,19]. The study by Papiez et al. show that improving movement quality throughout proper exercises decrease injury risk [20].

Physical performance is strongly associated with injuries. The review made by de la Motte et al. [13] indicates that weaker results in strength were associated with injuries among men and women. Disturbances in the balance of strength between antagonistic muscle groups may also promote injury [21]. If the flexibility level is low, the tissue will be more easily damaged. In the pre-season tests, football players presented smaller ranges of motion in the hip and knee joints they were more often injured during the season [22]. Literature reports indicate the relationship of lower limb power to injury [23]. It has been pointed out that a better ability to maintain balance is associated with fewer ankle injuries [24].

There is a relationship between physical performance and the quality of movement patterns [25]. Sannicandro et al. [23] showed an association between better FMS scores and higher power of lower limbs among professional footballers. Chimera et al. [26] showed strong links between flexibility and the quality of movement patterns and torso muscle strength. Similar observations were also made by Silva et al. [27], which showed the torso muscle strength as a factor determining the quality of movement patterns. Campa et al. [28] indicate a strong association between FMS score with repeated speed abilities and body composition, which shows morphological parameters are factors connected with movement patterns quality [29].

Given the interrelationship of physical performance and movement patterns, it is worth exploring the conditions of injuries through the prism of these factors. This is an important issue because, as previously indicated, injury conditions exist both concerning the quality of movement patterns and the physical performance [10,11,12,13,14].

There is a lack of studies on the determinants of injury that have simultaneously captured the quality of movement patterns and physical performance. Combined use of the two factors should show a stronger association with injury occurrence, implying more effective prevention injury methods. Lisman et al. [30] showed that combining the FMS score with endurance (3-mile run time) allowed a more accurate prediction of an injury among young soldiers.

The literature lacks a complex view of the determinants of injuries through the prism of movement patterns and physical performance; therefore, there is a strong need to explore this area. Accurate identification of factors connected with injury is crucial for prevention and implies effective injury-avoiding methods. It is necessary to answer: are the low-quality movement patterns associated with injury? If and which physical performance factors are linked with the injury? Additionally, do combined movement quality and physical performance estate, synergistic factors influence injury occurrence? Therefore, the main objective of this study is to examine if the movement patterns quality and physical performance simultaneously affected injury occurrence in young athletes? Specifically, we aimed to (1) examine the associations between movement quality and physical performance components with injury, (2) determine the minimal value of movement quality and associated physical performance factor for low risk of injury, (3) compare the number of injuries between groups differentiated based on the quality of movement patterns and physical fitness, and examine the interaction between these factors. The above observations can be helpful for coaches and athletes to effectively prevent injuries through shaping injury-related factors by appropriate training methods, leading to a reduction in the frequency of injuries.

## 2. Materials and Methods

### 2.1. Participants

The study sample was 176 young athletes (men and women). Participants were recruited from students of the Faculty of Physical Education and Sport. The study sample was 22.44 ± 1.64 years old. Men (N = 93) body height 1.81 ± 0.07 (cm), body weight 81.34 ± 9.48 (kg), BMI 24.94 ± 2.46 (kg/m^2^) Women (N = 83), body height 1.68 ± 0.08 (cm), body weight 60.80 ± 8.92 (kg), BMI 21.60 ± 2.47 (kg/m^2^). They have at least 10 years of experience in sport and are trained at least 3 times per week. From the whole study sample, 41 participants (23.29%) suffered at least one injury during the last 12 months. Subjects who suffered injuries 6 weeks directly before starting the study were excluded to avoid disturbances in conducted tests. All subjects were required to sign a written consent before participating in this study. They were informed in detail about the purpose, type, research methodology, and participation conditions. Participants could withdraw from the research at any time without giving any reason.

The research was carried out in the Biokinetics Research Laboratory of the University of Physical Education, which has a Quality Management System Certificate PN-EN ISO 9001: 2009 (Certificate Reg. No. PW-48606-10E). The Senate Research Ethics Committee approved the research at the University of Physical Education in Wrocław following the ethical requirements for human experiments under the Helsinki Declaration (consent number 16/2018).

### 2.2. Procedures

Measurement of height and weight-body height was measured to the nearest 0.1 cm and body weight to the nearest 0.1 kg. A SECA model 764 anthropometer was used, quality control number C-2070 (certificate 93/42 EEC, manufacturer: Seca GmbH & Co. KG. Germany). The body mass index-BMI (kg/m^2^) (body weight (kg)/body height (m^2^)) was calculated based on the obtained height and body mass values.

Injury data Injury History questionnaire (IHQ)—survey questionnaire regarding musculoskeletal system injuries suffered during physical activity. The IHQ consists of a simple question concerned about the number of injuries in a specified period. The survey was conducted in a supervised manner. The researcher was available to the respondents during the survey.

The IHQ reliability verification was assessed 7 days after participants took the IHQ; the survey was repeated among a randomly selected group of 56 people. The IHQ reliability was determined alpha-Cronbacha coefficient, which at level 0.836 indicated high reliability of IHQ [31]. The injury was defined as the onset of discomfort during physical activity that resulted in pain or discomfort within the musculoskeletal system, resulting in temporary limitation or complete inability to continue physical activity [32].

Quality of movement patterns-Functional Movement Screen (FMS)—The tests were performed with a standard FMS kit (Functional Movement Systems, Inc., Chatham, MA, USA). The FMS includes 7 single motor tasks: deep squat (DS), hurdle step (HS), IN-line lunge (IN-L), shoulder mobility (SM), active straight leg raise (ASLR), trunk stability push-up (TSPU), rotary stability (RS). According to the guidelines described for each test, every task was assessed on a scale of 0 to 3 [15]. When the examined person moves completely correctly, 3 points are awarded, 2 points when subjects move with visible compensation are awarded, and 1 point is awarded when the subject cannot complete the task. When a subject reports pain during a movement test, 0 points are awarded-regardless of the quality of the manifested movement pattern. The lower grade is considered for the overall grade for unilateral tests. Therefore, the maximum possible score is 21 points. The risk of injury increases significantly at and below 14 points [10].

Static strength of the upper limb-clamping a hand on the dynamometer—The clamping force of the right and left upper limbs was measured with an accuracy of 1 kg using a hydraulic dynamometer with an adjustable grip SAEHAN SH5001 (manufacturer: Saehan Corporation, South Korea). The examined person holds the arm lowered so that the arm, forearm, and hands do not touch the body. Clamping the dynamometer tightly to his hand, he performs the clamping of the hand with maximum force for about 2 s. Two attempts are made for each limb. The best result on both limbs is considered.

Flexibility–Sit and Reach Test—The equipment needed to perform the test is a table with the tabletop extending 15 cm above the sidewall on which the subject puts feet. There is a measuring tape parallel to the long axis on the countertop, with a scale from 0 to 50 cm, and a pointer loosely applied to move the tester’s hands during the test. The subject sits with the lower limbs straightened in the knee joints, putting his whole feet on the table’s sidewall. The subject makes a slope forward, keeping the knee extension straight, and moving the ruler on the counter as far as possible along the scale. Of the two attempts, a better result is considered. The measurement was made with an accuracy of 0.1 cm.

Strength of the lower limbs-long jump—The examined subject stands directly in front of the designated line and jumps, with a swing of the upper limbs landing on both legs. The length of the jump is measured from the reflection line on the rear edge of the heels. Two attempts are made. The better result is considered. The measurement was made with an accuracy of 0.5 cm.

Torso Muscle Strength-sit-ups—The attempt is to make as many sit-ups as possible in 30 s. The subject is placed on the floor with the lower limbs bent at the knee joints at an angle of 90 degrees. The feet are blocked in gymnastic ladders. The subject begins the test by lying down and hands folded at the nape of the neck. Throughout the test, he bends his torso with his elbows touching his knees. The test is carried out once. 

Static balance-ACCU SWAY stabilometric platform—with Balance Clince software (Advanced Mechanical Technology, Inc. [AMTI], Newton, MA, USA). The subject stands on the platform without shoes, with the upper limbs lowered along the torso. The task of the examined person is to maintain a stationary standing position for 30 s. The analyzed parameters were the distance along the center of gravity of the examined body and the field’s perimeter determined by the center of gravity path traveled during the measurement.

### 2.3. Statistical Analysis

The Cronbach’s alpha coefficient was calculated to determine the reliability of the IHQ survey [31]. The Shapiro-Wilk test tested the normality of the distribution of the analyzed variables. Means and standard deviations were calculated for data meeting distribution normality or median assumptions and standard errors for data not meeting normal distribution assumptions. Spearman’s rank correlation was calculated to examine the strength and direction of relationships between assessing the quality of movement patterns, physical performance, and injuries. The receiver operating characteristic curve allowed to dichotomously dividing the participants into groups according to the optimal point (cut-off point), indicating a high and low level of chosen factors correlated with injury occurrence. To indicate differences in injury frequency in the groups separated according to ROC curve cut-off point, aligned rank transform (ART) Anova was carried out, and Bonferroni post-hoc test was used. A non-parametric approach to factorial ART Anova enables the analysis of the interaction and the main effect [33]. The significance level was assumed in all statistical tests used, *α* = 0.05, which are highlighted in tables in bold. Statistica v13.0 from Statsoft Polska (Cracow, Poland) was used for statistical analyzes.

## 3. Results

Table 1 shows the mean results of the physical performance test. The median value of FMS overall score was 15. 

Table 2 shows the correlations between the physical performance tests and FMS assessment with injuries. Negative, statistically significant correlations were noted in movement patterns quality-overall FMS score, and the lower back and hamstring flexibility-sit-and-reach test with the number of injuries.

The ROC curve method was used to perform an aligned rank transform multivariate analysis of the determinants of injuries (ARTANOVA). The cut-off points values from which the frequency of injuries increases were determined for the quality of movement patterns and flexibility as the only factors correlating with injuries. This allowed for a dichotomous division of the subjects in terms of the frequency of injury.

The movement quality (FMS score) and flexibility (sit and reach) are including in the next parts of the analysis due to the shown association between them and injuries.

**Figure 1:**
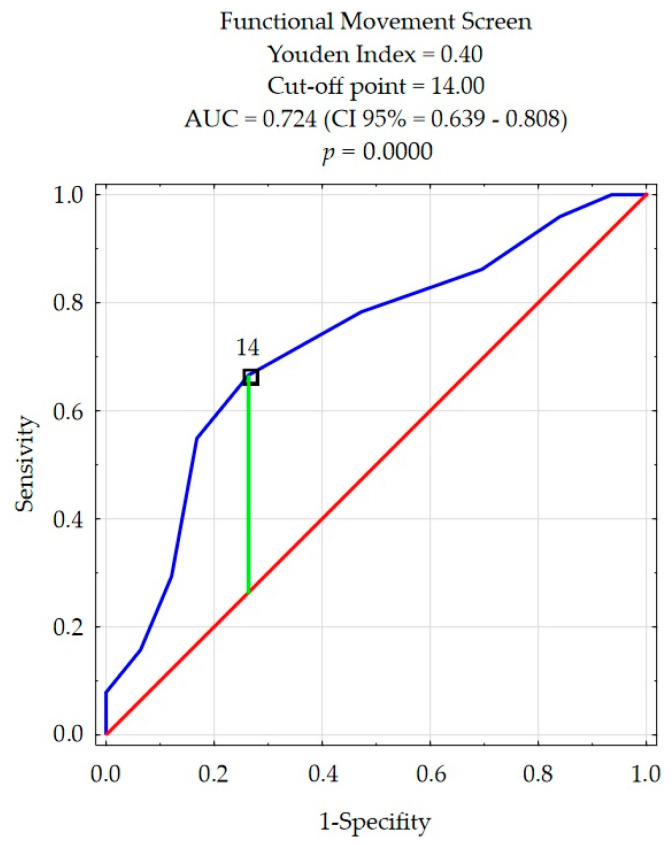
The cut-off point for quality movement patterns by frequency of injuries.

The FMS cut-off point value was 14 (Figure 1). On this basis, the participants were divided into groups of high-quality movement patterns (14 < FMS) in whom injuries occurred less frequently and subjects with low-quality movement patterns (14 ≥ FMS) in whom injuries occurred more often.

**Figure 2:**
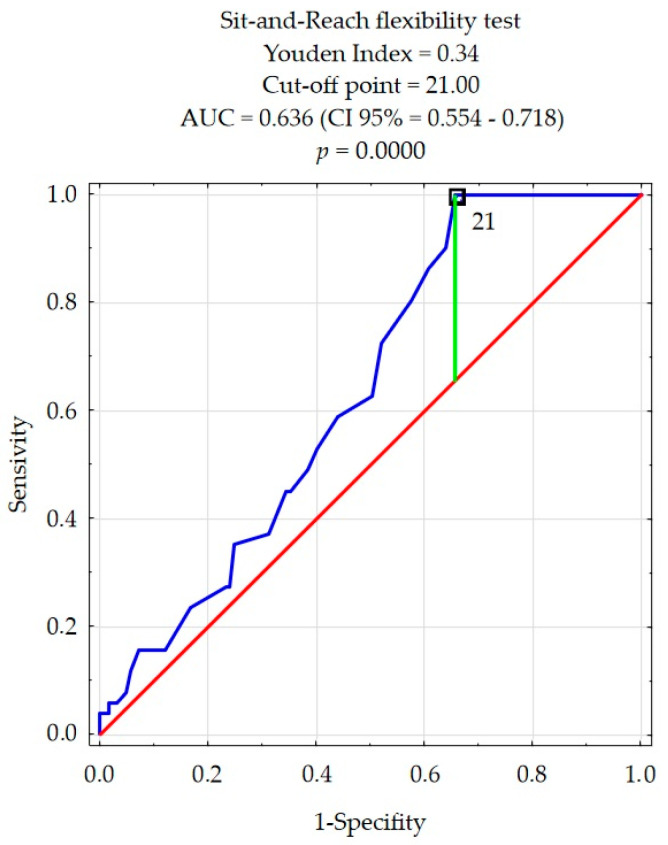
The cut-off point for the level of flexibility by frequency of injuries.

The cut-off point value for the level of flexibility lower back and hamstrings was 21 cm (Figure 2). On this basis, the participants were divided into groups with a high level of flexibility in whom injuries occurred less frequently (21 cm < Sit-and-reach score) and a low level of flexibility (21 cm ≥ Sit-and-reach score) in whom injuries occurred more often.

In Table 3, descriptive statistics of the ranks injuries data aligned before ranks calculation for groups separated from the study sample according to the cut-off point of movement quality (FMS score) and flexibility (Sit-and-reach test score).

The ART ANOVA analysis indicated diversity in injury frequency according to movement quality (*F* = 11.5361; *p* = 0.0008) and level of flexibility (*F* = 8.0514; *p* = 0.0050). However, there was no statistically significant interaction between relevant factors (*F* = 0.6465; *p* = 0.4224).

Table 4 indicates the diversity of injury frequency according to movement patterns quality and level of flexibility demonstrated by the post-hoc test. Athletes with low-quality movement patterns, combined with low-level flexibility (1), are more often injured than athletes from all other groups. There are no significant differences between groups 2, 3, and 4. 

## 4. Discussion

Injury prevention is one of the most significant challenges of modern sport. Accurate identification of factors contributing to injuries is vital in the appropriate selecting method of injury prevention. It is also important to take a multifactorial approach that considers many factors that may contribute to injuries. Minimizing the frequency of injuries is essential for young athletes, for whom an injury at this crucial time in their sport development can significantly inhibit or even disrupt their career [1,2]. Therefore, this study showed an injury connection with movement quality combined with physical performance. The complex view of injury occurrence considers some intrinsic factors as movement quality and physical performance could indicate an effective method in injury prevention.

Relationships between FMS and injuries have been shown in the literature [34]. However, some authors indicate that FMS assessment is not a sufficient tool in this area. They emphasize the need for a more comprehensive evaluation considering other factors, e.g., physical performance [6]. Chimera et al. [18] indicated weaker FMS scores among people who were more likely to suffer injuries regardless of sex. The study on a large group of female basketball players showed that those who had an FMS result below the cut-off point were more likely to suffer injuries than those above the indicated value [35]. Attwood et al. [19], in a study of a group of 277 rugby players, observed a higher frequency of injuries in players with low-quality movement patterns. In the study of Hotta et al. [36], it was indicated that FMS assessment could be useful in predicting injuries. Garrison et al. [11] show significant injury risk increase among collegiate athletes with low-quality movement patterns. Some different approaches towards injury occurrence among young athletes show Mokha et al. [37] which show the association between FMS asymmetries and single task scores with injury. The phenomenon of injuries is defined as complex and requiring multifactorial recognition. The complex approach may be helpful in decrease injury risk. It was suggested by Dorell et al. [38] which in the study among 256 young athletes, men and women show the FMS score could allow accurate prediction of injury. However, adding other factors to the analysis may increase correctness.

Physical performance is also emphasized as related to injuries [12,13,14]. Our research shows only statistically significant relationships between the sit and reach test, which measures lower back and hamstring flexibility. Many authors have pointed out the relationship between flexibility and injury [13]. Witvrouw et al. [39] showed that football players with less muscle flexibility, manifested by weaker joint movement ranges, were more likely to be injured than those who presented average values. Henderson et al. [40], among professional footballers, indicated players who suffered injuries more often had smaller ranges of motion in the joints, which is proved by a lower level of flexibility. Similar observation noted by Kobayashi et al. [41] in the study among collegiate athletes shows the association between decreased range of motion with injury. Sugiura et al. [42] proves that develop flexibility is an important factor in injury prevention. In the relevant study, there was no relationship between the sit-ups test result and injuries. It which was also observed by other authors [27]. The balance seems to be related to injuries [24]. However, in our study, there are no similar observations. The power of the lower limbs, in turn, may be associated with injuries. However, the selection of tests seems to be essential. Kodesh et al. [43] indicate a functional test as single-leg triple hop jump has relationships with injuries. 

In the light of reports indicating the relationships of quality of movement patterns and physical performance [25,28,29], few researchers have discussed the combined effects of these factors on injury occurrence. The Lehr et al. [44] study indicated that a more comprehensive assessment in injury occurrence might be more effective in diagnosis and prevention than single-factor analysis. Therefore, identification of a few factors and connection between them may let to avoid an injury. Especially worth exploration is combining intrinsic factors as a movement patterns quality or a physical performance shaped during the training. Therefore, its level is the effect of exercise, modulated by a proper method [5].

Our research indicated that flexibility and quality of movement patterns correlated with injury occurrence and the combination of this factor synergistic influence on frequency injuries. Lisman et al. [30] show among 874 men relationships of FMS assessment with injuries. Additionally, using fitness tests, they showed that a weaker 3-mile run combined with low-quality movement patterns is more likely to be associated with injuries. This observation suggests endurance as a factor connected with the injury. The sit-ups test used in the same study-as in the own study-was not associated with injuries. A similar attempt to assess injury conditions was made by Kodesh et al. [43]. However, in their research, was showed relationships of injuries only with motor skills in terms of endurance, without indicating relationships between FMS scores and injuries in a group of 145 female soldiers. 

## 5. Conclusions

Movement quality and flexibility are associated with injury. For the FMS test score, the minimal value of low injury risk is 14 points, whereas, for a sit-and reach-test, 21 cm. However, these values should be used with caution. Athletes with low-quality movement patterns combined with low flexibility of the lower back and hamstrings are the most often injured than other groups. However, only high-quality movement patterns seem to be enough factors in the reduction in injury frequency. The interaction between movement quality and flexibility is insignificant what indicates they are additive. Development of high-quality movement patterns and improving the lower back and hamstring flexibility level may reduce the prevalence of injuries. Coaches and athletes should strive to improve the quality of movement patterns and flexibility to prevent injuries. There is a need to introduce exercises in training to develop movement quality and flexibility. Comprehensive work in this area is suggested to increase the positive results. The relevant observation indicates the possible direction in protection from injury among young athletes. Further study should focus on the verification and development of methods and tools improving the quality of movement patterns and flexibility and finding combinations and interactions between injury factors for more accurate diagnosis and prediction.

## Figures and Tables

**Table 1:** Descriptive statistics of physical performance test results.

Factor	Mean	SD	CI −95%	CI +95%
Hand grip [N/kg]	47.80	13.16	45.84	49.75
Long jump [cm]	200.10	34.71	194.92	205.28
Sit-ups [reps/30 s]	28.74	5.25	27.96	29.52
Sit and reach [sm]	16.15	9.05	14.80	17.50
Balance-area circle [cm^2^]	2.24	1.39	2.01	2.47
Balance-path length [cm]	38.86	7.82	37.58	40.14

**Table 2:** Spearman correlation for injuries and physical performance tests scores, FMS overall score (*p* < 0.05).

Factor	Injury	*p* Value
FMS Overall score	−0.3360	0.0000
Hand grip	0.0760	0.3782
Long jump	0.0373	0.8168
Sit-ups	−0.0181	0.8062
Sit-and-reach	−0.2075	0.0076
Balance-area circle	0.0701	0.4404
Balance-path length	−0.0775	0.3009

**Table 3:** Descriptive statistics of the ranks injuries aligned before ranks calculation.

Group	N	Mean	SD	CI −95%	CI +95%
1-LQ movement patterns; LL flexibilty	55	111.69	44.57	99.64	123.74
2-LQ movement patterns; HL flexibilty	12	86.00	42.53	58.98	113.02
3-HQ movement patterns; LL flexibilty	71	82.05	37.49	73.18	90.93
4-HQ movement patterns; HL flexibilty	38	67.71	20.61	60.94	74.49

Abbreviations: HQ movement patterns- high-quality movement patterns, FMS score < 14; HL flexibility—high-level flexibility, sit-and-reach score < 21; LQ movement patterns—Low-quality movement patterns 14 ≥ FMSscore; LL flexibility—low-level flexibility 21 ≥ sit-and-reach score.

**Table 4:** Detailed comparisons between four groups—post hoc tests results.

Groups	1–2	1–3	1–4	2–3	2–4	3–4
Value	0.0327	0.0019	0.0000	0.7362	0.1421	0.0583

Abbreviations: 1—Low-quality movement patterns; Low level of flexibility. 2—Low-quality movement patterns; High level of flexibility. 3—High-quality movement patterns; Low level of flexibility. 4—High-quality movement patterns; High level of flexibility.

## Data Availability

The datasets used and/or analyzed during the current study are available from the corresponding author on reasonable request.
